# Saphenous vein graft interposition following aggressive endarterectomy for right coronary artery: experience with 32 cases

**DOI:** 10.1186/1749-8090-8-S1-O171

**Published:** 2013-09-11

**Authors:** Y Besir, O Gokalp, H Cakır, E Celik, H İner, A Gurbuz

**Affiliations:** 1 Cardiovascular Surgery Department, Izmir Atatürk Education and Research Hospital, Izmir, Turkey

## 

Increasingly more diffuse and complex atherosclerotic lesions are encountered in coronary bypass surgery. Although coronary endarterectomy has been performed for a long time, it possesses risks of mortality and morbidity. For LAD techniques such as closure with saphenous patch and patch + anastomosis with LIMA are used following endarterectomy. In this case series, we aimed to present our experience with saphenous interpositions that we performed or were compelled to perform following endarterectomy carried out on the trunk of RCA. Of 554 patients operated with primary coronary surgery by the same surgical team between 2008 and 2012, 32 underwent right coronary endarterectomy. Median sternotomy was performed and standard aortobicaval cannulation + antegrade + retrograde blood cardioplegia were used in all surgeries. Saphenous vein grafts were used in all RCA anastomoses. Care was taken to excise the plaque as a whole with an endarterectomy blade after right coronary arteriotomy. All of the aggressive endarterectomy applications performed in those 32 patients were against trunk lesions of the RCA. The length of the plaques excised with endarterectomy ranged between 6-10 cm. After completion of endarterectomy, a saphenous interposition was performed at the trunk region of the right coronary artery and then a saphenous vein bypass was carried out distal to the interposition as deemed necessary after a check with coronary probe. All operations were completed uneventfully and patients were taken to the ICU after the operation. They were discharged between postoperative days 5-7.Only 4 patients developed postoperative atrial fibrillation, which were converted back to normal sinus rhythm by medical therapy. No sign of ischemia was observed in the RCA region in scintigraphic examination performed 12 months after the operation. We suggest that saphenous vein graft interposition performed after endarterectomy for the trunk of RCA is safely applicable, as trunk region of RCA does not have any side branch.

